# Current Status and Associated Factors of Treatment Burden in Stroke Patients: Mixed‐Methods Research With a Convergent Parallel Design

**DOI:** 10.1155/nrp/9873977

**Published:** 2026-02-16

**Authors:** Rui Zhou, Saisai Xu, Yue Sun, Hongmei Shen, Huimin Yang, Xinran Zhou, Ziqi Xu, Zhicheng Zhang, Chengbiao Lu, Guodong Wang

**Affiliations:** ^1^ School of Nursing, Henan Medical University, Xinxiang, 453000, Henan, China; ^2^ Department of Emergency Medicine, The General Hospital of Western Theater Command, Chengdu, 610036, Sichuan, China; ^3^ The Fourth Affiliated Hospital of Henan Medical University, Xinxiang, 453000, Henan, China; ^4^ School of Medical Sciences, Henan Medical University, Xinxiang, 453000, Henan, China

**Keywords:** associated factors, cumulative complexity model, stroke, treatment burden

## Abstract

**Aim:**

To investigate the current status of treatment burden in stroke patients and analyze its associated factors, thereby providing a theoretical basis for clinical interventions.

**Design:**

This study used a convergent parallel mixed‐methods design.

**Methods:**

For the quantitative phase, a questionnaire survey was conducted among hospitalized stroke patients using convenience sampling from April to October 2024. Univariate analysis, correlation analysis, and multiple regression analysis were used to identify the associated factors of treatment burden. For the qualitative phase, purposive sampling was adopted, and the Colaizzi seven‐step method with NVivo 11 software was used to analyze the specific manifestations and experiences of treatment burden in stroke patients. The quantitative and qualitative results were finally integrated and triangulated for joint presentation.

**Results:**

A total of 237 valid questionnaires were retrieved. Multiple linear indicated that depression (*β* ^′^ = 0.431, *p* < 0.001), self‐management efficacy (*β* ^′^ = −0.235, *p* = 0.047), age (*β* ^′^ = −0.213, *p* = 0.001), objective support (*β* ^′^ = −0.171, *p* = 0.001), and diet control (*β* ^′^ = −0.163, *p* = 0.001) were the main associated factors of treatment burden of stroke patients. Seventeen stroke patients were interviewed for the qualitative phase, from which 8 themes and 27 subthemes were extracted. Integration of quantitative and qualitative results yielded a comprehensive, multidimensional understanding of treatment burden in stroke patients.

**Conclusion:**

Treatment burden in stroke patients is associated with multiple factors, involving psychological, behavioral, and social support dimensions. This finding emphasizes the necessity for healthcare professionals to adopt a holistic approach to stroke patient care.

**Implications for Clinical Practice and Patient Care:**

This study suggests that clinical practice should strengthen psychological interventions for stroke patients, improve their self‐efficacy, provide individualized guidance, and refine support systems, so as to effectively alleviate treatment burden.

## 1. Introduction

Stroke remains the second leading cause of death worldwide and the third leading cause of death and disability [1]. Stroke is characterized by high disability and high recurrence, and rehabilitation and secondary prevention are of great significance in reducing the disability and recurrence rates of stroke patients [2]. However, the lengthy rehabilitation and treatment process poses a significant challenge to stroke patients’ own capabilities. Patients may experience varying degrees of treatment burden during this process. Treatment burden (TB) is the sum of time, energy, emotion, and financial costs during long‐term disease management, and higher TB is associated with lower treatment adherence and, ultimately, poorer quality of life for stroke patients [3]. In China, while a nationwide stroke care network has been established, disparities in resource allocation and service capacity persist [4]. This systemic unevenness may compromise the accessibility and quality of rehabilitation services, thereby potentially exacerbating the TB on patients. Therefore, a systematic assessment of TB in stroke patients is of considerable importance for identifying service delivery gaps and informing patient‐centered quality improvement in care.

## 2. Background

Stroke patients not only have to cope with the therapeutic management of a chronic disease but also need to undergo rehabilitation and exercise for sequelae. Combined with the long‐term treatment cycle, these factors put them at a higher risk of high TB compared to patients with other chronic diseases [5]. Healthcare professionals often overlook TB; they tend to focus more on patients’ treatment tasks and biomedical status, while patients are more concerned about their level of social support [6]. In clinical practice, some healthcare professionals tend to adopt intensive treatment to optimize patients’ clinical outcomes without fully considering their TB. This may lead to excessive treatment intensity, leaving patients physically and psychologically exhausted, which in turn exacerbates the difficulty of disease management and reduces patients’ quality of life [7].

**FIGURE 1:**
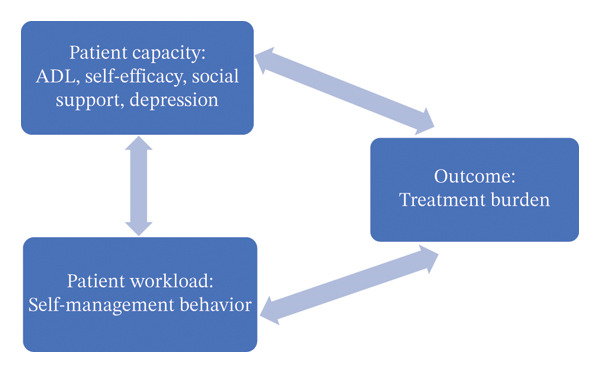
Conceptual framework of research on associated factors of TB in stroke patients.

TB is closely associated with patient‐centered care. When healthcare professionals adopt a patient‐centered perspective, they can identify the problems and difficulties patients encounter during treatment, provide timely assistance, adjust treatment plans, and reduce the energy patients invest. This ensures that the patients’ treatment workload does not exceed their coping capacity, thereby improving treatment adherence and willingness to participate in continuous treatment and reducing adverse prognosis [8]. The cumulative complexity model (CuCoM) was proposed by Shippee et al. [9]. This model posits that the deterioration of patients’ health status is primarily caused by an imbalance between their medical tasks and actual coping capacity. It emphasizes patient‐level mechanisms and highlights the dynamic relationship between the workload patients’ face during treatment and their coping capacity. The model comprises two core components: patient capacity, which refers to personal resources such as physical/mental function, symptoms, finances, and social support; and patient workload, which encompasses the behavioral demands generated by the illness, such as scheduling/attending appointments and medication management. Therefore, this study develops a conceptual framework diagram (see Figure 1) based on the CuCoM, from the perspective of patient capacity and patient workload. Meanwhile, a mixed‐methods (MM) study design is adopted; the integration of quantitative and qualitative findings can comprehensively explore the associated factors of TB in stroke patients, aiming to provide a basis for the implementation of optimized stroke management strategies to improve patients’ treatment experience and quality of life.

## 3. Method

### 3.1. Aim

To investigate the current status of TB in stroke patients, explore the associated factors of TB, and reveal the subjective experiences and behavioral patterns of stroke patients related to TB. Additionally, to integrate quantitative and qualitative research findings, thereby enhancing the explanatory power of the research conclusions and providing empirical support for clinical practice and subsequent related research.

### 3.2. Design

This study adopts a convergent parallel MM design to explore the associated factors of TB in stroke patients [10]. The reporting of this MM study adhered to the GRAMMS criteria (see Supporting Information 1).

### 3.3. Data Collection

#### 3.3.1. Quantitative Data

Participants were recruited using convenience sampling. All patients who presented to the Departments of Neurology, Neurosurgery, and Rehabilitation Medicine at a public tertiary hospital in northern China between April and October 2024, and who met the following inclusion/exclusion criteria, were invited to participate in this study. The inclusion criteria were as follows: aged 18 years or older; at least 2 weeks poststroke onset; diagnosed with ischemic or hemorrhagic stroke and in a clinically stable condition; possessing normal comprehension ability and the capacity to express personal intentions independently; and willing to participate in this study and able to provide written informed consent. Patients with mental illness or other severe conditions (e.g., malignant tumors, heart failure, and respiratory failure) that could interfere with the study outcomes were excluded from the study. All included patients completed the self‐designed general information questionnaire, the Treatment Burden Questionnaire, the Stroke Self‐Efficacy Questionnaire (SSEQ), the Social Support Rating Scale (SSRS), the 9‐item Patient Health Questionnaire (PHQ‐9), and the Stroke Self‐Management Behavior Scale (SSMBS).

The sample size required for the quantitative study in this research was determined in accordance with the Kendall criterion. The dependent variable was TB, and 28 independent variables were included: 16 items from the general information questionnaire, two dimensions from the SSEQ, three dimensions from the SSRS, one dimension from the PHQ‐9, and six dimensions from the SSMBS. According to the standard that the sample size should be five to ten times the number of independent variables, the sample size was calculated to be 140–280. Considering a 20% nonresponse rate, the required sample size was adjusted to 168–336. Finally, 253 questionnaires were distributed, with 237 valid responses (valid response rate 93.68%), which met the sample size requirement.

General information questionnaire: nine items in the demographic‐related information section—age, gender, education level, marital status, place of residence, work status, medical payment method, per capita monthly household income, and primary caregiver; and seven items in the disease‐related information section—the type of stroke, duration of illness, number of sequelae, comorbidities with chronic diseases, number of types of oral medications taken per day, and number of stroke episodes, activities of daily living (ADL) degree. The degree of ADL was assessed by the Barthel index (BI) scale, 100 being self‐care, 60–99 being mildly dependent, 41–59 being moderately dependent, and ≤ 40 being severely dependent. The scale has Cronbach’s alpha coefficient of 0.929 and has been widely used [11].

Treatment Burden Questionnaire (TBQ): developed by Tran et al. [12] to evaluate the TB borne by patients with chronic diseases. Zhang et al. [13] Chineseized and applied it to stroke patients, including three dimensions with 15 entries, of which “self‐management” includes —one to six entries, “seeking medical care” includes 7–11 entries, and “lifestyle” includes 12–15 entries, using Likert 11 scale, the higher the total score, the higher the TB of the patient, the total Cronbach’s alpha coefficient of the scale is 0.824. In the current sample, the Cronbach *α* coefficient of the total scale was 0.795.

SSEQ: initially developed by Jones et al. [14] and later translated into Chinese by Li et al. [15] to assess the level of rehabilitation confidence of stroke patients. The scale consists of two dimensions, in which the self‐management efficacy dimension consists of five entries and the ADLs efficacy dimension consists of six entries. The scale was rated on a 10‐point Likert scale from “not at all confident” to “very confident,” with values ranging from 0 to 10. The Cronbach alpha coefficient for this scale is 0.969, and the Cronbach alpha coefficient for this study is 0.980.

SSRS: developed by Xiao [16] to measure the level of social support received by an individual. It consists of three dimensions: subjective support (Items 1, 3, 4, and 5), objective support (Items 2, 6, and 7), and social support utilization (Items 8, 9, and 10), and the higher the total score, the higher the level of social support received by the individual. This scale has a total Cronbach’s alpha coefficient of 0.920 and has been widely used in Chinese stroke patients [17]. Cronbach’s alpha coefficient in this study was 0.801.

PHQ‐9: one of the internationally recognized self‐assessment screening tools for depression. The scale consists of nine entries in one dimension, with each entry scoring from 0 to 3, and the higher the total score, the more severe the depression. Currently, this scale has been widely used in stroke patients [18]. The Cronbach alpha coefficient in this study was 0.878.

The SSMBS: compiled by Chen et al. [19], it was divided into six dimensions of dietary control, emotional management, medication adherence, rehabilitation exercise, weight control, and smoking and alcohol cessation, with a total of 20 entries. A Likert five‐point scale was used, with a score range of 0–4 for each entry. Higher total scores indicated better patient self‐management behavior. The Cronbach alpha coefficient of the questionnaire was 0.813. In the smoking cessation and alcohol restriction dimension, in order to reasonably differentiate the workload in self‐management between patients who do not smoke and/or do not drink alcohol and those who do smoke and/or drink alcohol, a mean substitution method was used, and baseline scores were given to patients who do not smoke and/or do not drink alcohol for the adherence to cessation and adherence to the restriction of alcohol entries in order to avoid their scores on this dimension was scored as 0, resulting in an underestimation of total workload. The Cronbach alpha coefficient in this study was 0.764.

#### 3.3.2. Qualitative Data

Qualitative data were collected through face‐to‐face semistructured interviews. The interviews were conducted in a hospital conference room to ensure participants could express their genuine thoughts in a relatively quiet and comfortable environment. All interview data collection was undertaken by the researcher, who had previously engaged in the daily care of the interviewees to understand their real‐life situations and establish a trusting relationship with them. One‐on‐one interviews were conducted with each patient, and all sessions were audio‐recorded, lasting 20–30 min per interview. Patient recruitment continued until “data saturation” was achieved. A combination of purposive sampling and maximum variation sampling was adopted to recruit participants from the pool of patients who had participated in the quantitative phase. From the pool of survey respondents, a purposive sampling strategy incorporating maximum variation was employed to select interviewees based on demographic characteristics (e.g., age and educational level), TB level (TBQ score ≥ 75), and disease‐related characteristics (e.g., stroke type and disease duration). This approach ensured diversity across key dimensions, thereby capturing a wide range of illness experiences and burden perceptions. A semistructured interview guide was formulated based on the CuCoM and revised after pilot interviews with three stroke patients (not included in the final analysis) to enhance its clarity and relevance. The interview guide covered two core dimensions aligned with the model: (1) patient capacity dimension: stress and challenges encountered during treatment, difficulties in understanding and implementing physician‐prescribed treatment plans, and support from family or friends regarding treatment; and (2) patient workload dimension: difficulties in managing daily disease‐related tasks (e.g., medication adherence, rehabilitation training, medical appointment scheduling), time and effort invested in these tasks, and changes and challenges in life following stroke onset. Additionally, patients were given the opportunity to discuss any other TB‐related issues not addressed in the interview. Interview transcripts were returned to the participants within 48 h for the verification of their accuracy and completeness. A staged thematic combing method was adopted: after conducting 10 and 15 interviews, respectively, the research team coded and analyzed the interview data using NVivo 11 software. No new themes or subthemes emerged after the 15th interview. To confirm saturation, two additional interviews were conducted (a total of 17 interviews), and the analysis results showed no new information was added. Thus, data saturation was achieved, and data collection was terminated.

### 3.4. Data Analysis

The results of the quantitative survey were entered into the questionnaire using EpiData 3.1 Chinese version, and a two‐person checking method was adopted to ensure the accuracy of the data, and 20% of the data were randomly double‐checked to ensure the accuracy of the information entry. Statistical analysis was performed using IBM SPSS Statistics 25.0 software. Measurement information used mean and standard deviation to describe the basic characteristics; count information used the frequency and percentage to describe the basic characteristics. One‐way analysis of variance was performed using two independent samples *t* test or ANOVA analysis. Pearson correlation coefficient/Spearman correlation coefficient was used to analyze correlation. Multiple linear regression analysis was used to determine the main associated factors of TB by taking the statistically significant variables in the single‐factor analysis and correlation analysis as independent variables. A *p* value < 0.05 was considered statistically significant for all statistical tests.

Qualitative interview data were transcribed verbatim within 24 h using Microsoft Word 2021. Reflexive accounts were written immediately following each interview to enhance rigor and explore potential bias. Each reflexive account included personal thoughts on the major ideas emerging from the participant’s narrative, as well as reflections on the researcher–interviewee interaction and rapport that may have influenced data collection. The research team reviewed these notes repeatedly throughout data collection to refine interview techniques for the subsequent sessions. Transcripts were returned to participants within 48 h for member checking to ensure completeness and accuracy. Following multiple readings of the transcripts, the research team conducted thematic analysis using Colaizzi’s seven‐step method combined with NVivo 11 software for coding and management. Data collection and analysis occurred simultaneously, and recruitment ceased once data saturation was achieved. Saturation was determined by assessing code meaning rather than counting codes—defined as gaining a full understanding of an issue or code with no new facets identified. Saturation was reached after 15 interviews, with two additional interviews conducted to confirm no new information emerged. During analysis, notes were taken to document participants’ statements, associations, and preliminary interpretations. Reflexive accounts were re‐read, and descriptive notes were transformed into broader categories or themes involving a higher level of abstraction. These steps were repeated for each transcript: themes from the previous transcript helped orient the analysis of the next, while leaving space for new ideas. Regular debriefing with coauthors involving the discussion of multiple views enabled analytical triangulation. Themes were further refined, and the authenticity and credibility of the data were enhanced by reorganizing and synthesizing the themes after conducting 10 and 15 interviews, respectively. Two integration strategies were adopted to the integration of quantitative and qualitative results: ① triangulation integration, where quantitative statistical results and qualitative thematic findings were cross‐validated to enhance conclusion credibility and ② sequential integration, where qualitative insights were used to interpret quantitative trends. A joint display table was constructed to visually present the confirmation, complementarity, and expansion relationships between the two data strands, providing a holistic understanding of TB in stroke patients.

### 3.5. Rigorous

In this study, quantitative and qualitative research were carried out simultaneously, and the data of the two studies were thoroughly mined. The quantitative and qualitative findings were presented separately, and then, the results were comprehensively integrated to determine the relationship between the quantitative and qualitative results in this study and to comprehensively analyze the factors influencing the generation of TB in stroke patients.

### 3.6. Ethical Consideration

The study was approved by the Ethics Committee of Xinxiang Medical University (XYLL‐20240273). The researcher explained the purpose, significance, and possible benefits and risks of the study to each participant. Surveys or interviews were conducted after ensuring that participants fully understood the relevant information. All collected questionnaires and interviews were numbered and filed, and strict confidentiality measures were taken to ensure that participants’ personal information was not leaked.

## 4. Results

### 4.1. Quantitative Results

#### 4.1.1. Univariate Analysis of General Information and TB in Stroke Patients

A total of 253 questionnaires were distributed, and 237 questionnaires were validly returned, with a valid return rate of 93.68%; the youngest of the stroke patients participating in the study was 19 years old, and the oldest was 90 years old, with a mean age of (58.30 ± 12.48) years old; of these, 154 (65.0%) were male and 83 (35.0%) were female. The results of univariate analysis showed statistically significant differences in the TB of stroke patients in terms of age, work status, per capita household family income, medical payment method, primary caregiver, type of stroke, duration of illness, number of sequelae, number of comorbidities with chronic diseases, number of types of oral medications, and degree of ADL (*p* < 0.05). Full information is provided in Table 1.

**TABLE 1:** Results of univariate analysis of general information and TB for patients with stroke (*N* = 237).

Variable	Groups	Cases/composition ratio (%)	Scores	*t*/*F* value	*p*
Gender	Male	154 (65.0)	66.50 ± 12.15	0.711^①^	0.478
	Female	83 (35.0)	65.22 ± 15.10		

Age	< 45	26 (11.0)	75.42 ± 7.23	17.588^②^	< 0.001
	45∼	100 (42.2)	70.06 ± 12.71		
	60∼	90 (38.0)	60.32 ± 11.26		
	≥ 75	21 (8.9)	59.91 ± 16.01		

Marital status	Unmarried/divorced/widowed	25 (10.5)	64.96 ± 15.61	−0.435^①^	0.664
	Married	212 (89.5)	66.18 ± 12.97		

Place of residence	City	111 (46.8)	64.60 ± 12.76	−1.584^①^	0.114
	Rural	126 (53.2)	67.33 ± 13.57		

Education level	Primary and lower	49 (20.7)	65.76 ± 13.33	0.832^②^	0.477
	Junior middle school	105 (44.3)	67.15 ± 13.35		
	High school or junior college	59 (24.9)	65.78 ± 11.96		
	College and above	24 (10.1)	62.50 ± 15.53		

Work status	Unemployed	118 (49.8)	66.66 ± 13.07	7.482^②^	< 0.001
	Sick leave	19 (8.0)	73.68 ± 9.98		
	On duty	36 (15.2)	69.86 ± 12.45		
	Retired	64 (27.0)	60.52 ± 12.93		

Per capita monthly household income (CNY)	≤ 2000	67 (28.3)	67.24 ± 12.20	4.631^②^	0.004
	2001∼	51 (21.5)	68.71 ± 12.40		
	3001∼	66 (27.8)	67.41 ± 12.69		
	> 4000	53 (22.4)	60.30 ± 14.58		

Medical payment method	Medical insurance/public funds	230 (97.0)	65.73 ± 13.27	−4.563^②^	0.002
	Self‐funded	7 (3.0)	76.71 ± 5.94		

Type of stroke	Ischemic	135 (57.0)	61.83 ± 12.98	18.753^②^	< 0.001
	Hemorrhagic	88 (37.1)	72.08 ± 11.40		
	Both	14 (5.9)	68.86 ± 11.58		

Primary caregiver	Parents	10 (4.2)	75.60 ± 5.17	4.111^②^	0.003
	Spouse	135 (57.0)	66.59 ± 13.51		
	Child	53 (22.4)	60.83 ± 12.07		
	Carer	27 (11.4)	69.52 ± 12.57		
	Relative or other	12 (5.1)	67.25 ± 14.26		

Duration of illness (months)	< 6	151 (63.7)	67.34 ± 13.24	2.006^①^	0.046
	≥ 6	86 (36.3)	63.78 ± 13.01		

Number of stroke episodes	1 time	153 (58.4)	67.22 ± 13.47	1.733^②^	0.179
	2 times	68 (26.0)	63.71 ± 12.97		
	≥ 3 times	16 (6.1)	64.88 ± 11.24		

Number of sequelae	None	22 (9.3)	53.14 ± 13.60	12.423^②^	< 0.001
	1 type	91 (38.4)	64.47 ± 12.74		
	2 types	84 (35.4)	68.37 ± 12.01		
	≥ 3 types	40 (16.9)	71.86 ± 11.53		

Comorbidities with chronic diseases	None	53 (22.4)	68.93 ± 14.04	2.951^②^	0.033
	1 type	96 (40.5)	65.18 ± 13.12		
	2 types	62 (26.2)	63.15 ± 12.62		
	≥ 3 types	26 (11.0)	70.35 ± 11.89		

Number of types of oral medications taken per day	≤ 2 types	35 (14.3)	60.91 ± 15.44	3.149^②^	0.045
	3∼5 types	130 (54.9)	66.89 ± 12.67		
	≥ 6 types	72 (30.4)	67.03 ± 12.71		

ADL degree	Severely dependent	61 (25.7)	70.07 ± 11.79	11.381^②^	< 0.001
	Moderately dependent	53 (22.4)	70.83 ± 12.06		
	Mildly dependent	85 (35.9)	63.98 ± 12.22		
	Self‐care	38 (16.0)	57.58 ± 14.36		

*Note:* ①: *t* value; ②: *F* value.

#### 4.1.2. Current Status of the TB for Stroke Patients

The TBQ scores of stroke patients in this study ranged from 30 to 96 (66.05 ± 13.23), and the mean score of the entries was (4.40 ± 0.88). The mean scores of seeking medical care and lifestyle dimensions were greater than 4.4, and only the self‐management dimension had lower mean scores, suggesting that the perceived TB of stroke patients in this dimension is low (Table 2).

**TABLE 2:** Current status of TB scores in stroke patients (*N* = 237, $\overset{¯}{x}$ ± *S*).

Variable	Entries	Highest score	Lowest score	Average score	Average score of entries
TBQ	15	96	30	66.05 ± 13.23	4.40 ± 0.88
Self‐management	6	40	10	20.90 ± 5.06	3.48 ± 0.84
Seeking medical care	5	37	11	22.91 ± 5.72	4.58 ± 1.14
Lifestyle	4	33	8	22.24 ± 5.61	5.56 ± 1.40

#### 4.1.3. Correlation Analysis

The results of the correlation analysis showed that TBQ in stroke patients was negatively correlated with SSEQ, SSMBS, and SSRS, with correlation coefficients of −0.395, −0.522, and −0.348, respectively, all *p* < 0.001, and positively correlated with PHQ‐9, with a correlation coefficient of 0.639, *p* < 0.001 (Table 3).

**TABLE 3:** Correlation analysis of TB in patients with stroke.

Variable	*r*/*ρ*	*p* value
SSEQ	−0.395^①^	< 0.001
The self‐management efficacy	−0.409^①^	< 0.001
The activities of daily living efficacy	−0.359^①^	< 0.001
SSMBS	−0.522^①^	< 0.001
Dietary control	−0.137^①^	0.036
Medication adherence	−0.274^①^	< 0.001
Emotional management	−0.608^①^	< 0.001
Rehabilitation exercise	−0.363^①^	< 0.001
Smoking and alcohol cessation	−0.145^②^	0.025
Weight control	−0.051^①^	0.434
SSRS	−0.348^②^	< 0.001
Subjective support	−0.462^②^	0.094
Objective support	−0.462^①^	< 0.001
Social support utilization	−0.445^①^	< 0.001
PHQ‐9	0.639^①^	< 0.001

*Note:* ①: *r*; ②: *ρ*.

#### 4.1.4. Multifactorial Analysis of TB in Stroke Patients

TBQ of stroke patients was used as the dependent variable, and the statistically significant variables in the univariate and correlation analyses were selected as independent variables with *α*_in_ = 0.05 and *α*_out_ = 0.10. Multiple linear regression analysis was performed. The independent variable assignments are shown in Supporting Information 2. The covariance diagnostics showed that the tolerances were all greater than 0.1, and the VIFs were all less than 10, indicating that there was no problem of multicollinearity among the independent variables. The results of the multiple linear regression showed that depression, self‐management efficacy, age, objective support, and dietary control were influential factors in the TB of stroke patients, explaining 59.9% of the total variance (see Table 4; the complete table is available in Supporting information 3).

**TABLE 4:** Multiple linear regression analysis of TB in stroke patients.

Variable	*β*	SE	*β* ^′^	*t*	*p*	TOL	VIF
*β*_0_	102.517	9.703		10.565	< 0.001		
PHQ‐9	1.309	0.209	0.431	6.250	< 0.001	0.369	2.713
The self‐management efficacy	−0.286	0.144	−0.235	−1.995	0.047	0.126	7.910
Age	−3.551	1.005	−0.213	−3.532	0.001	0.481	2.081
Objective support	−1.702	0.515	−0.171	−3.302	0.001	0.651	1.537
Dietary control	−2.008	0.607	−0.163	−3.307	0.001	0.720	1.389

*Note: F* = 13.225, *p* < 0.001; *R* ^2^ = 0.648; adjusted *R* ^2^ = 0.599.

### 4.2. Qualitative Results

#### 4.2.1. General Information on Qualitative Research Participants

In this study, qualitative interviews were conducted with a total of 17 stroke patients (nine males and eight females), aged between 21 and 68 years, coded as N1 to 17. See Table 5 for details.

**TABLE 5:** General information on patients participating in the interview (*N* = 17).

Number	Gender	Age	Marital status	Education level	Work status	Type of stroke
N1	Male	58	Married	Junior middle school	Unemployed	Hemorrhagic
N2	Male	35	Married	High school or junior college	On duty	Ischemic
N3	Female	45	Married	High school or junior college	Sick leave	Hemorrhagic
N4	Female	61	Married	Junior middle school	Unemployed	Ischemic
N5	Female	27	Married	College and above	Unemployed	Hemorrhagic
N6	Male	68	Married	Primary and lower	Unemployed	Both
N7	Female	53	Married	Junior middle school	Unemployed	Ischemic
N8	Male	63	Married	Junior middle school	Retired	Ischemic
N9	Male	21	Unmarried	High school or junior college	Unemployed	Hemorrhagic
N10	Female	62	Married	Primary and lower	Retired	Ischemic
N11	Male	32	Divorced	College and above	Unemployed	Ischemic
N12	Female	37	Married	High school or junior college	Unemployed	Hemorrhagic
N13	Female	60	Married	High school or junior college	Retired	Ischemic
N14	Male	61	Married	Primary and lower	Unemployed	Both
N15	Male	55	Married	Junior middle school	Unemployed	Hemorrhagic
N16	Female	52	Married	High school or junior college	Sick leave	Ischemic
N17	Male	57	Married	Junior middle school	Sick leave	Ischemic

#### 4.2.2. Coding Framework

All qualitative data were managed and coded using NVivo 11, which was used for thematic organization, code merging, and visual analysis of interview transcripts.

A three‐level coding framework was constructed based on the CuCoM. The first‐level coding corresponds to the two core components of the model: “patient capacity” and “patient workload.” The second‐level coding derived eight main themes from these two core categories, and the third‐level coding further subdivided each main theme into 27 subthemes.

#### 4.2.3. Interview Results

In this study, the interview data were coded and categorized to coalesce eight themes:

 Theme 1: imbalance of psychological status
 Subtheme ① high psychological pressure. Most patients’ lives have been seriously affected because of the stroke. Patients said that they were under great psychological pressure, their physical condition suddenly deteriorated, they lost their ability to work and faced with great economic pressure, and they felt deeply guilty and blamed themselves.
 N1: “Being ill is hard on one’s heart. When I was healthy, I could work anywhere, but now that I can do nothing, I feel like a burden.” N3: “It’s a bad mood because I’m hospitalized.”
 Subtheme ② thinking confusedly. Patients have pessimistic associations with their illness, and their thinking state is greatly influenced by emotions, so they are pessimistic about the future.
 N12: “Especially at night, I am more prone to rambling thoughts, worrying about my family’s health and fearing that I will drag them down in the future.” N15: “Love to ramble on about messy, strange things, always thinking about my family, wife, children, siblings, parents, I want to see them too much, seeing one less than the other.”
 Subtheme ③ negative emotional distress. Patients are uncertain about their future recovery, and there are fears and worries in their hearts, which easily lead to negative emotions such as anxiety and depression.
 N3: “Recently, I have a quick temper, I love to lose my temper with people, and my mood is sometimes good and sometimes bad.” N17: “Life is too tiring, now I don’t want to live, too tired, I’m sorry to my parents for leaving, but the pressure on me is too much.”
 Theme 2: Limitation of physical functional status.
 Subtheme ① limb mobility limitation. Affected by the severity of the disease, the motor function of patients may be impaired to varying degrees, leading to the decline of daily living ability and even inability to take care of themselves.
 N4: “My upper limbs and legs, I feel like I can’t use my strength, and although daily activities can be partially accomplished, these still affect my life.” N7: “Even basic things like eating and washing up require help and I find it particularly difficult.”
 Subtheme ② discomfort caused by pain. The physical discomfort caused by pain directly affects daily life. Persistent or repeated pain will aggravate the psychological pressure of patients and make them resist rehabilitation training.
 N3: “Every time it hurts so much that I don’t want to practice, I’m afraid to go to practice, and I always feel like I can’t stand the pain when I practice.” N10: “I can’t sleep at night either, my head swells and hurts from time to time, and I feel uncomfortable how I sleep.”
 Subtheme ③ difficulty swallowing and eating. Some patients will suffer from different degrees of swallowing function damage after stroke, manifested as eating and medication difficulties, which has a great impact on the daily diet of patients.
 N6: “Now I have to vomit when I take a few pills, before I used to be able to eat everything fine, like rice and vegetables can be swallowed.” N13: “Eating used to be enjoyable, now every meal is like a task, sometimes I don’t want to eat halfway through, and my family has to make the food extra soft or make a paste to make it work.”
 Theme 3: Multiple impacts of financial ability on patients.
 Subtheme ① high cost of treatment. Patients need to pay higher costs during treatment and worry about the economic burden of disease recurrence and long‐term treatment, and the family economic situation is difficult to support future treatment costs.
 N2: “It costs a lot of money every time I am hospitalized for treatment and rehab, and I know that my family bears a lot of the burden for me.” N10: “I just don’t have the money, I need to spend 20,000 RMB for one visit, it’s a lot of pressure.”
 Subtheme ② decline in income levels. Patients are unable to continue working due to illness, resulting in a decline in family income levels, while living expenses remain, putting great financial pressure on them.
 N2: “I myself am the breadwinner of my family, and now that I can’t earn enough money, my children can barely keep up with their school expenses.” N15: “I can’t work when I’m sick. I used to earn a few thousand a month, but now even the basic expenses of my family are a problem.”
 Subtheme ③ concerns about future work capacity. Patients are anxious about their future ability to work and worried that they will not be able to do their previous jobs after recovery, and their financial independence will be threatened.
 N2: “The most troublesome thing is the work, originally can still do some physical work, now no one wants to use me, afraid that I suddenly fainted and had an accident, even some simple part‐time jobs can’t be found.” N17: “I don’t think I can work again even after recovery. Construction sites already refuse workers over 58, and with my health condition, I definitely won’t be hired.”
 Theme 4: impacts of social support on patients’ lives
 Subtheme ① the need for emotional companionship. Emotional support from family and friends, especially when depressed, is important for psychological recovery.
 N6: “Well (whimpering), (family members) came a total of one time (crying).” N8: “Some friends and neighbors visit me often after I got sick. They help me with chores and chat with me, which warms my heart.” N11: “They came to visit me from time to time or called to say hello, which made me feel that even though I was sick, I was not isolated.”
 Subtheme ② practical care and resource support. Life‐care support (e.g., diet and cleaning) and resource assistance from society, family, and friends reduce the likelihood of patients interrupting treatment.
 N3: “My family encourages me to stick to treatment and rehabilitation. They also help financially—money is transferred directly to me when I need it.” N9: “My family and friends have been incredibly helpful. My family takes care of me around the clock—they bring me food, chat with me, give me massages, and encourage me to exercise.”
 Subtheme ③ transfer of family responsibilities and caregiving pressure. After the patient falls ill, family members need to take on additional care responsibilities, not only to take care of their daily life, but also to deal with family affairs, so that the patient feels guilty.
 N2: “My family helps take care of me, but my work situation really bothers me. I used to be quite busy, so now that I’m forced to a sudden stop, I feel useless.” N3: “My kids are young and no one helps them with homework, so their grades have dropped. My parents are old and need care too, but I can’t do anything for them now.”
 Subtheme ④ internal struggles in the process of being supported. Although patients feel the care and help from family and friends, many patients will feel inferiority and guilt because they cannot live independently and think that they have become a burden to their families and society.
 N11: “I sometimes feel inferior, like I’m drifting apart from my friends who are healthy and working.” N12: “My family never complains, which touches me deeply, but I feel guilty—I know I’m dragging them down.”
 Theme 5: disturbances during treatment.
 Subtheme ① discomfort from injections. In the process of cooperating with the treatment, the pain, fear, and discomfort caused by injection put a burden on the patient’s body and mind.
 N7: “I’m actually scared of injections, especially before each shot. I pretend not to be, but seeing the needle still makes me nervous. I’ve gotten used to it after so many times, but the fear never fully goes away.” N14: “Injections are unavoidable, but I’m afraid the nurse might miss the vein and move the needle around. I hate the pain.”
 Subtheme ② side effects and uncertainty of drugs. The side effects of drugs and the uncertainty of drug efficacy make patients feel confused and worried, especially about the variety, long‐term use, and effectiveness of drugs.
 N6: “I took 5 or 6 kinds of medicine during my first 12 day hospitalization. I didn’t ask the doctor how to take them after discharge, and the hospital’s medicine made me weak, so I stopped taking it. Now I only take traditional Chinese medicine once a day, which I’ve been doing for 2 months.” N8: “There are so many types of medicine, and taking them long‐term upsets my stomach. I worry about the burden on my liver and kidneys, but I have to take them as the doctor prescribed.”
 Subtheme ③ fatigue due to frequent checkups. Frequent examination, especially long‐term monitoring process, makes patients feel uncomfortable and tired. In the case of mobility difficulties, patients need to rely on the assistance of others, further increasing the burden.
 N2: “The biggest difficulty is the unclear cause. Initially, I was treated for cerebral thrombosis, but it didn’t work. Then, I was told it was a heart issue and underwent a closure procedure. After each treatment, the doctor would say I should be fine, yet the problem recurred. Numerous tests were done, but a clear answer never emerged, and this uncertainty made me very anxious.” N10: “The tests were really frequent and felt a bit overwhelming. Several tubes of blood are drawn, and my body is already weak, so I feel drained of energy after each blood draw.”
 Subtheme ④ the ordeal of prolonged treatment. The long‐term treatment process has brought continuous psychological suffering and emotional stress to patients. Patients will feel physically and mentally exhausted when faced with repeated treatment and hospitalization, especially when the treatment has no obvious effect.
 N3: “The rehabilitation department doesn’t have a psychiatrist. It’s hard to stay here with nothing to do—I wish I could talk to a psychiatrist when I’m feeling down.” N7: “Every time I am hospitalized, I need to adjust my daily living arrangement, which makes me feel a bit troublesome, but I can only try to cooperate in order to cure my illness. Sometimes it also feels like an ordeal to go to the doctor, and I hope that my health will get better soon and I won’t need to be hospitalized and treated so often.”
 Theme 6: physical and mental burdens of rehabilitative exercises.
 Subtheme ① physical fatigue and discomfort. Rehabilitation exercise often leads to physical fatigue and discomfort; especially when the exercise intensity is high or the project schedule is tight, the patient is easy to feel physical deficiency and discomfort, which increases the physical burden in the rehabilitation process and may affect the patient’s willingness to continue exercising.
 N3: “The last rehabilitation exercise is walking while holding the railing—I just can’t do it. I don’t know how to walk properly, and I’m sweating profusely after trying. I even want to kneel down from exhaustion.” N15: “Although the workout itself is not difficult, my body gets tired after each training session, especially when multiple events are centrally scheduled, and sometimes I feel like my body can’t keep up with the pace.”
 Subtheme ② insufficient motivation due to psychological resistance. Although the patient knows that rehabilitation exercise is essential, the patient has resistance to exercise due to physical discomfort. During exercise, the patient sometimes feels very tired, weak, and resistant, resulting in reduced exercise compliance.
 N7: “I don’t want to stick to the rehabilitation exercise once I am tired, I can’t even stick to it for 10 min” N16: “I can accept taking medicine and doing tests, but rehabilitation exercises are tormenting. Sometimes I really don’t want to do them, but I know I have to.”
 Subtheme ③ frustration caused by the slow progress of rehabilitation. Although the patient can feel some improvement in physical ability, the progress of recovery does not meet expectations, and the patient may feel frustrated and have doubts about the effect of treatment. It is difficult for the patient to maintain continuous recovery motivation, resulting in depression.
 N3: “Progress is slower, I hope I get better soon myself, it’s been over 2 months since I got sick and my left leg has gone from not being able to walk to only being able to walk a few steps now, I think the rehabilitation progress is too slow.” N7: “When I did rehabilitation exercises, I saw that my progress was so slow, so I didn’t want to continue even more. But my family encourages me again, saying that it’s one step at a time and slowly, but sometimes it’s annoying to hear that.”
 Subtheme ④ doubt about the effect of rehabilitation. The slow pace of recovery leads to doubts about the effectiveness of treatment and anxiety. Patients worry that the intensity of exercise is not enough, or the treatment method is not appropriate, and they worry that the recovery effect will not achieve the expected goal.
 N3: “I just hope that the hospital will get in some better instruments (I feel that the current equipment is not advanced enough) so that the patients can get better as soon as possible.” N7: “However, sometimes my physical condition is unstable (my body gets better and worse at times), which also makes me doubt the effectiveness of the exercise, worrying whether it is not strong enough or in the wrong direction.”
 Theme 7: difficulties of execution in daily life.
 Subtheme ① discomfort in controlling diet. Patients usually actively or passively restrict their diet and avoid eating high‐salt and high‐fat foods. When faced with others enjoying tempting food, patients may feel psychologically lost.
 N2: “I used to eat heavy, now I eat lighter, sometimes I still don’t get used to it.” N15: “Although the doctor didn’t have any special requirements for diet, I know myself that I have to pay attention to what I eat in general, I am too fat. In fact, sometimes when I look at my family members eating their favorite things, I still can’t help but crave them, but for the sake of my health, I can only restrain myself.”
 Subtheme ② difficulty in performing self‐monitoring. Patients lack experience with self‐monitoring and often do not develop the habit of regularly measuring health indicators such as blood pressure and blood sugar, and patients may be confused about these new tasks, have difficulty adhering to them, and worry about the accuracy of measurement results.
 N4: “The healthcare workers did teach me how to use the electronic blood pressure monitor, and every time they demonstrated it, I slowly started to be able to operate it by myself. However, when I take the measurement myself, I am still not too confident about the result, especially if the value is high, I will start to think nonsense and become nervous.” N14: “My doctor suggested that I buy an electronic blood pressure monitor, and I considered it, but I was worried about whether I would be able to keep it up with long‐term use. It’s not easy for us country people to develop this habit.”
 Subtheme ③ challenges of quitting smoking and drinking. As part of treatment, many patients face a greater psychological burden at the beginning. Patients are forced to quit smoking and alcohol at the advice of doctors and family members, but they still feel difficult and cause psychological struggles in actual life situations and dependence.
 N6: “The doctor said smoking and drinking are bad for blood vessels and will hinder my recovery. When I was first hospitalized, I secretly wanted to smoke a cigarette to satisfy my craving, but I didn’t dare after the doctor’s warning. I’ve quit completely now, but I still think about it sometimes.” N14: “Quitting alcohol is relatively easy, I basically didn’t have the chance to drink after being hospitalized, but it’s really a bit difficult to quit smoking, and now I’m still okay with touching my pocket to look for cigarettes.”
 Theme 8: experience in the process of seeking medical treatment
 Subtheme ① inconvenience during medical procedures. Due to mobility difficulties caused by his illness, the patient sought medical treatment with the help of his family or others. Especially during relapse or hospitalization, the patient’s mobility difficulties make every medical visit a difficult task.
 N1: “The biggest headache for me during my hospitalization was the inconvenience of daily life. A trip to the bathroom was especially difficult for me, and I had to rely on nurses or family members every time, which was (embarrassing).” N13: “The process of going to the doctor was very frustrating for me, especially since I had to be hospitalized every time I had a relapse. It’s especially hard to get around now that I have no dexterity in my hands and feet.”
 Subtheme ② small difficulties in the medical procedure. While medical institutions have relatively streamlined procedures for administrative formalities and treatment processes, bringing certain convenience to patients and alleviating some unnecessary burdens, patients still face certain difficulties.
 N2: “It’s not too troublesome now. If you don’t know where to go, just ask the doctor – they’ll tell you. It’s much more convenient than before. But I still don’t know how to handle things that need to be done at the community.” N7: “The procedure is simple, I give you a time, and the next day I go and do it, but I have to go every few days (it’s quite troublesome).”
 Subtheme ③ insufficient support from doctors and nurses. Treatment support, assistance, and communication provided by doctors and nurses are an important part of the patient’s treatment process. Although patients feel supported, sometimes patients still feel inadequate.
 N3: “I told the doctor, and the doctor just said to hold on, only if you hold on can you get better.” N12: “The doctor explains things very clearly and the nurse helps with everything, I think it’s good, I’m just afraid that people will think I’m troublesome.”

### 4.3. Integration of Results

The integration of factors influencing the burden of care for stroke patients in terms of two dimensions, patient capacity and patient workload, was guided by the CuCoM, and inferences about the results were made. The conformity evaluation between quantitative and qualitative results is confirmation, complementarity, and expansion [20]. See Table 6.

**TABLE 6:** Joint presentation of findings of quantitative and qualitative research results and inferences from the results.

	Quantitative findings	Qualitative findings	Inference of results
Patient capacity	Relationship between psychological factors and TB: Depression level was a major influence on TB (*p* < 0.001) and positively correlated with TB (*p* < 0.001); self‐management efficacy was a major influence on TB (*p* = 0.047); SSEQ was negatively correlated with TB (*p* < 0.001); and activities of daily living efficacy was negatively correlated with TB (*p* < 0.001).	Theme 1: Imbalance of psychological status Theme 6: Physical and mental burdens of rehabilitative exercises (three subthemes, “insufficient motivation due to psychological resistance,” “frustration caused by the slow progress of rehabilitation,” and “doubt about the effect of rehabilitation,” describe psychological changes resulting from rehabilitation exercises.)	Expansion: The quantitative results indicate the relationship between psychological factors and TB. The qualitative results specify the patients’ perceived psychological state during the treatment process, concretizing reduced self‐efficacy into the cognitive‐behavioral experiences of “psychological resistance,” “frustration,” and “doubt about effectiveness,” while also suggesting the presence of emotions such as anxiety.
	Relationship between physical status and TB: Degree of ADL was an influential factor in TB (*p* < 0.001); number of sequelae was an influential factor in TB (*p* < 0.001); and age was a major influential factor in TB (*p* < 0.001), a negative relationship.	Theme 2: Limitation of physical functional status	Expansion: Quantitative results clarify the relationship between physical condition and TB, demonstrating a negative relationship between TB and age. The qualitative results explore the actual impact and experience of physical limitations on patients, describing difficulties with pain and swallowing. The quantitative and qualitative findings form an extension of each other.
	Relationship between economic status and TB: Work status was an influencing factor on TB (*p* < 0.001); per capita monthly household income was an influencing factor on TB (*p* = 0.004); and medical payment method was an influencing factor on TB (*p* = 0.002).	Theme 3: Multiple impacts of financial ability on patients	Confirmation: Quantitative results indicated that TB varied by work status and per capita household income. The qualitative findings reveal the specific manifestations of economic factors in TB from the patient’s perspective, indicating the sources of economic pressure, which is consistent with the results of the quantitative study
	The relationship between social support and TB: Objective support was a major influence on TB (*p* = 0.001) and was negatively associated with TB (*p* < 0.001); social support was negatively associated with TB (*p* < 0.001); social support utilization was negatively associated with TB (*p* < 0.001); primary caregiver was an influence on TB (*p* = 0.003); subjective support was associated with TB (*p* = 0.094); marital status and treatment burden (*p* = 0.094).	Theme 4: Impacts of social support on patients’ lives	Complementarity: Quantitative results demonstrated the relationship between social support and TB. The qualitative results reveal the multifaceted impact of social support on patients’ lives, specifically describing the role of tangible subjective support such as emotional companionship and practical care for patients, which complements the quantitative results.

Patient workload	Relationship between the treatment process and TB: Duration of illness was an influencing factor on TB (*p* = 0.046); Comorbidities with chronic diseases was an influencing factor on TB (*p* = 0.033); number of types of oral medications taken per day was an influencing factor on TB (*p* = 0.045); medication adherence was negatively correlated with TB (*p* < 0.001); number of stroke episodes and TB (*p* = 0.179).	Theme 5: Disturbances during treatment	Complementarity: Quantitative results clarify the relationship between treatment process‐related factors and TB. The qualitative results validate and describe the actual experience of treatment process–related factors in patients’ lives. The qualitative study complemented the quantitative results by demonstrating the actual impact of relapse on patients’ TB through patients’ experiences.
	Relationship between rehabilitation exercise and TB: Rehabilitation exercise was negatively associated with TB (*p* < 0.001)	Theme 6: Physical and mental burdens of rehabilitative exercises	Expansion: Quantitative results demonstrated the relationship between rehabilitative exercise and TB. Qualitative results extend the specific manifestations and further enrich the understanding of the impact of rehabilitative exercise on TB.
	Relationship between lifestyle management and TB: Dietary control was a major influence on TB (*p* = 0.001) and was negatively associated with TB (*p* = 0.036); smoking and alcohol cessation were negatively associated with TB (*p* = 0.025); and emotional management was negatively associated with TB (*p* < 0.001).	Theme 7: Difficulties of execution in daily life	Expansion: Quantitative results demonstrate the relationship between self‐management of daily life and TB. Qualitative results analyze the specific challenges of patients in self‐management of daily life and expand on the fact that difficulties in self‐monitoring are also relevant associated factors.
	Relationship between seeking medical treatment and TB: seeking medical care dimension score (22.91 ± 5.72), mean score of entries (4.58 ± 1.14).	Theme 8: Experience in the process of seeking medical treatment	Confirmation: Quantitative outcomes reflect the overall burden and experience of the patient’s healthcare process. The qualitative results indicate the actual experience of the patient’s healthcare process and are consistent with the quantitative results.

## 5. Discussion

In this study, the TB score for stroke patients was (66.05 ± 13.23), indicating a moderate level of burden and highlighting the need for healthcare professionals to prioritize TB management in this population. This score was slightly higher than the (62.45 ± 24.35) reported in a related study [21], which may be attributed to regional differences in socioeconomic development, healthcare resource accessibility, and rehabilitation service models. Among the three dimensions of TB, the lifestyle adjustment dimension yielded the highest mean score, reflecting the substantial workload imposed on stroke patients during rehabilitation—including dietary modifications, adherence to exercise regimens, and adjustments to interpersonal relationships. In contrast, the self‐management dimension had the lowest mean score, which may stem from insufficient disease‐specific knowledge, limited self‐management skills, cognitive impairments, economic constraints, or inadequate social support [22]. These factors reduce patients’ capacity to execute daily health management tasks, leading to lower perceived TB in this dimension due to compromised task engagement rather than reduced workload.

### 5.1. Impact of Patient Capacity on TB in Stroke Patients

The CuCoM posits that TB arises when the demands of treatment (workload) exceed a patient’s ability to cope (capacity) [8]. Psychological capacity, a core component of overall patient capacity, plays a pivotal role in TB formation and exacerbation. Stroke‐induced psychological stress can amplify perceived workload by reducing patients’ cognitive and emotional resources to engage with rehabilitation exercises, thereby eroding self‐efficacy and treatment adherence—key mediators of capacity–workload balance. This study indicates that depression is a primary factor influencing the TB of stroke patients, which is consistent with the findings from a previous investigation of elderly patients with chronic comorbidities [23]. Poststroke depression (PSD), a prevalent psychological disorder affecting 27.5%–62.5% of patients in the acute phase [24], directly impairs psychological capacity: Depressed patients exhibit significantly lower self‐efficacy compared to nondepressed peers, with a strong negative correlation between depression severity and self‐efficacy [25]. This reduction in psychological capacity widens the gap between treatment workload (daily medication, rehabilitation training) and coping ability, exacerbating perceived TB. To address this, healthcare systems should integrate routine screening for psychological disorders into stroke care pathways, enabling early detection of mood changes and timely implementation of targeted interventions. Evidence‐based approaches such as acceptance and commitment therapy (ACT) and positive thinking training [26] can enhance psychological capacity by fostering adaptive coping strategies. For stroke patients diagnosed with PSD, intervention should be actively combined with psychologists and comprehensive means such as medication and physical therapy should be used according to the patient’s condition in order to alleviate depressive symptoms and optimize the rehabilitation effect [27].

Physical capacity, another critical dimension of patient capacity, is shaped by ADL function and the number of poststroke sequelae. Dysfunction increases patients’ reliance on daily care and rehabilitation services, which in turn elevates their investment in time, energy, and finances [28]. Age is a primary factor influencing the TB of stroke patients, showing a negative association, which is consistent with the findings of Tahsin et al. [29] in chronic disease populations. Younger patients perceive heavier TB, possibly due to the need to juggle professional and family roles and higher rehabilitation expectations, which predispose them to anxiety and feelings of powerlessness, whereas older patients have relatively adapted to living with a slow illness and are more receptive to treatment [29]. Qualitative findings complement these quantitative results, identifying pain and dysphagia as key barriers to physical capacity—limiting functional recovery, increasing emotional workload, and reducing treatment adherence [30, 31]. Therefore, healthcare professionals should monitor patients’ physical functional status, develop individualized rehabilitation plans, strengthen pain management and dysphagia interventions, and enhance patients’ cognitive appraisal and coping capacity for functional changes, thereby reducing their perceived treatment workload.

In terms of economic factors, low‐income patients and those with self‐paid medical expenses face higher perceived TB, as do employed or on‐sick‐leave patients compared to unemployed or retired individuals—consistent with the findings of Khojah et al. [32] in a chronic comorbid patient population. Financial toxicity (FT), which refers to the economic burden that patients suffer from disease‐related medical expenditures and reduced income and its negative impact on psychological and quality of life, is currently used in stroke populations [33]. FT reduces patients’ treatment adherence. To cut short‐term healthcare costs, patients may forgo essential medications, opt for less efficacious alternative therapies, or even discontinue treatment entirely—ultimately prolonging recovery cycles and increasing long‐term medical expenditures [34]. To alleviate the perceived TB in stroke patients caused by economic factors, healthcare professionals should assess patients’ financial status, identify FT in a timely manner, and implement targeted economic interventions based on financial navigation to help patients optimize resource utilization. Additionally, patients’ affordability should be taken into consideration when formulating treatment plans [35]. At the policy level, expanding medical insurance coverage and social assistance systems can reduce direct medical costs, alleviating the economic workload [36].

Social support, as a crucial moderator of patients’ capacity, is negatively correlated with TB. Objective support serves as the primary protective factor. Although subjective support lacked statistical significance in the quantitative study, patients in qualitative interviews indicated that the emotional companionship of family members and friends plays an important role in rehabilitation. This phenomenon may stem from two reasons: first, patients’ ambivalent experiences of support (as illustrated in the subtheme “internal struggles in the process of being supported”), where emotional needs and guilt offset each other, and their interaction may have weakened the statistical association between subjective support and TB; and second, the standardized items of the SSRS struggle to fully capture dynamic and context‐specific emotional experiences, whereas qualitative interviews are more sensitive to revealing such subjective feelings. By supplementing patients’ psychological, physical, and economic resources, social support enhances their overall capacity, thereby reducing the effective workload of treatment [37]. Furthermore, qualitative data reveal a more complex correlation: When the primary caregivers are patients’ parents, patients may experience feelings of self‐reproach and guilt due to responsibility transfer and emotional conflicts, which undermines their motivation for rehabilitation [38]. In clinical practice, stroke patients should be encouraged to leverage social support while enhancing their personal capacity, so as to reduce over‐reliance on caregivers and improve rehabilitation outcomes.

### 5.2. Effect of Patient Workload on TB in Stroke Patients

Patient workload, defined by the CuCoM framework as the cumulative demands of medical care, rehabilitation, self‐management, and lifestyle adjustments, is a key determinant of TB. Patients with a short duration of illness report higher TB, as they have not yet adapted to the sudden increase in workload including coping with acute functional impairments and reconstructing daily routines. Poor medication adherence, a wide range of medications, and a high number of comorbidities may increase TB. Qualitative findings elaborate on this, noting that some patients have anxiety due to medication side effects and uncertainty of efficacy, which in turn affects adherence. In addition, physical and mental exhaustion due to frequent injections, examinations, and prolonged treatment was frequently mentioned by patients, reflecting the role of excessive medical manipulation in the formation of TB. Therefore, for the therapeutic management of stroke patients, attention should be paid to the adaptation process of patients in the early stages of the disease, and personalized health guidance and psychological support should be provided [39]. At the same time, timely optimization of medication regimens, such as medication reconciliation (Med⁃Rec) and other methods, reduces unnecessary medical manipulation and examination, and enhances patients’ confidence in long‐term medication [40].

As a core component of treatment workload, rehabilitation exercise is negatively correlated with TB, indicating that structured exercise can enhance physical capacity, reduce care dependency, and thereby improve the capacity–workload balance. However, qualitative data reveal significant barriers associated with rehabilitation workload: Particularly in the early stages of rehabilitation, patients frequently report physical fatigue, pain, and discomfort, which limit their training tolerance [41]. Psychological workload also plays a role: resistance to exercise, lack of motivation, and frustration from slow progress erode adherence and exacerbate TB [42, 43]. Rehabilitation exercises for stroke patients are multifaceted, and in order to improve patients’ adherence to rehabilitation and reduce the difficulties brought about by the rehabilitation process, it can be combined with the multidisciplinary team (MDT) model, which integrates medical, rehabilitation, nursing, and other resources to provide patients with a full range of rehabilitation services [44]. In terms of improving physical ability, the results of Lee et al. [45] systematic evaluation showed that task‐oriented training (TOT) has an important value, and its advantage lies in the combination of the actual life needs of patients so that the rehabilitation training is more targeted and functional, and it can be combined with the individualization of patients, combined with other rehabilitation measures, to improve the patient’s physical function and recovery effect.

The present study found that lifestyle modifications (including dietary control, smoking and alcohol cessation, and emotional management) were negatively correlated with TB, indicating that successful adaptation to these changes can alleviate the burden. The qualitative results further revealed that patients often face both psychological and behavioral dilemmas in the execution of life behaviors. For example, although dietary control can reduce the burden, some patients have a psychological gap when facing dietary temptations, and for those who passively accept restrictions, they may resist due to a lack of understanding [46]. Although regular health monitoring is important, some patients have difficulty adhering to it due to operational barriers or anxiety about the results, delaying intervention for their condition [47]. Smoking cessation and alcohol restriction, on the other hand, is often accompanied by withdrawal reactions due to the addictive nature, which aggravates anxiety and affects behavioral persistence [48]. To mitigate the workload associated with lifestyle modifications, comprehensive intervention strategies should be implemented. Lau et al. [49] showed that self‐management intervention programs based on theories (social cognitive theory, family systems theory, etc.) are more likely to enhance health behaviors in stroke patients. Meal planning programs driven by artificial intelligence (AI) can generate personalized healthy meal plans based on health status, personal preferences, etc., thus enhancing adherence to dietary management in stroke patients [50].

### 5.3. Balance Analysis of Stroke Patient Capacity and Patient Workload

Guided by the CuCoM framework, regression analysis and qualitative findings collectively demonstrate that TB in stroke patients arises from an imbalance between capacity and workload: high negative affect, low self‐efficacy, and younger age reduce capacity, while objective support and dietary control enhance capacity and reduce perceived workload. Qualitative data further reveal that patients often struggle to translate self‐management requirements into action, as their actual capacity is mismatched with the demands of treatment workload—leading to increased burden. This aligns with the core tenet of the CuCoM framework, which emphasizes that TB is not inherent to treatment but emerges from the interaction between patient capacity and treatment demands. Minimally disruptive medicine (MDM) and patient‐centered care (PCC) offer complementary approaches to optimizing the capacity–workload balance, emphasizing that treatment should minimize disruption to patients’ lives while ensuring efficacy [8, 51]. To alleviate TB in stroke patients, interventions should focus on two key pillars: enhancing capacity and rationalizing workload. Based on the study findings, three targeted strategies are proposed: (1) strengthen capacity building—implement routine psychological screening and interventions to address negative emotions and enhance psychological capacity. Provide structured self‐management training to improve disease knowledge, skills, and self‐efficacy, enabling patients to cope more effectively with treatment demands; (2) optimize patient workload—develop individualized rehabilitation and treatment plans that align with patients’ age, physical function, and psychological state. For younger patients, set realistic recovery goals to reduce psychological workload; for patients with comorbidities, simplify medication regimens and minimize redundant tests to reduce cognitive and physical workload. (3) Enhance support systems—strengthen objective social support (e.g., financial assistance) and promote effective support utilization to supplement patients’ inherent capacity. Encourage family and community involvement to reduce the workload of daily activities and rehabilitation, while avoiding role conflicts that may increase psychological burden.

## 6. Strengths and Limitations

This study constructs a research framework based on the CuCoM, analyzes the association between patient capacity, workload, and TB from a dual perspective of patient capacity and workload, reveals the complex mechanisms underlying TB formation, and provides theoretical references for the subsequent studies. Meanwhile, the convergent MM design was adopted, combining quantitative analysis and qualitative interviews to ensure that the research results are more realistic and instructive.

The limitations of this study are as follows: (1) The study adopted a single‐center, convenience sampling design and only recruited hospitalized stroke patients. Regional variations in cultural backgrounds and economic development levels may restrict the generalizability of the findings to the broader stroke population. (2) Quantitative research mainly relied on questionnaire surveys, which may be subject to self‐report bias. (3) The SSMBS primarily focused on the execution of behaviors, lacking an assessment of the time and energy invested by patients during implementation, which limits the accuracy of workload evaluation.

## 7. Suggestions for Future Research

(1) With the longitudinal study design, the changing trend of TB was dynamically tracked to understand the changes of TB in stroke patients in different periods; (2) improve the design of patient workload questionnaire to measure patient workload more accurately; and (3) make an empirical study on relevant measures to reduce the TB (e.g., telemedicine and AI‐driven self‐management tools) to provide scientific basis.

## 8. Conclusion

Based on the CuCoM, this MM study systematically assessed TB in Chinese stroke patients and explored the associated factors of TB from the dual perspectives of patient capacity and treatment workload, thereby not only generating comprehensive empirical evidence on the moderate level of TB in this population and its key associated factors (including depression, self‐management efficacy, age, objective social support, and dietary control) to inform clinical practice, but also validating the applicability of the CuCoM framework in the Chinese stroke population by demonstrating that the framework can effectively elucidate TB as a product of the dynamic interaction between patient capacity and treatment workload. Qualitative findings further elaborated on the specific manifestations of TB in stroke patients, enriching the understanding of the pathways underlying TB development and complementing the quantitative results to form a holistic insight into TB among Chinese stroke patients. Drawing on the integrated quantitative and qualitative results, this study proposes targeted strategies to alleviate TB in stroke patients, including strengthening psychological interventions, optimizing self‐management support, delivering individualized rehabilitation guidance, and enhancing social and family support, which aim to improve patients’ coping capacity and optimize the distribution of treatment workload, ultimately providing actionable guidance for clinical TB management in stroke patients and laying a foundation for subsequent related research.

## Author Contributions

Made substantial contributions to conception and design, or acquisition of data, or analysis and interpretation of data: Rui Zhou, Saisai Xu, and Yue Sun. Data collection and interpretation: Hongmei Shen. Interpreting data and writing: Saisai Xu, Huimin Yang, and Xinran Zhou. Interpretation of data: Ziqi Xu and Zhicheng Zhang. Involved in drafting the manuscript or revising it critically for important intellectual content: Chengbiao Lu and Guodong Wang.

## Funding

The authors declare that the research, creation, and/or publication of this article have not received any financial support.

## Conflicts of Interest

The authors declare no conflicts of interest.

## Supporting Information

Supporting information 1: Good reporting of a mixed‐methods study (GRAMMS) checklist.

Supporting information 2: Description of independent variable assignment.

Supporting information 3: Multiple linear regression analysis of TB in stroke patients.

## Supporting information


**Supporting Information** Additional supporting information can be found online in the Supporting Information section.

## Data Availability

The data of the results of this study can be obtained from the author. Due to privacy or ethical constraints, these data will not be made public.
