# Supplementary material for: Current Status and Associated Factors of Treatment Burden in Stroke Patients: Mixed‐Methods Research With a Convergent Parallel Design
Source: Nurs Res Pract. 2026 Feb 16;2026:9873977. doi: 10.1155/nrp/9873977 (PMC12910179; doi:10.1155/nrp/9873977)
Supplement: Supplementary file 1 — Supporting Information Additional supporting information can be found online in the Supporting Information section. [file NRP-2026-9873977-s001.docx]

Supplementary material 1 Good Reporting of a Mixed Methods Study (GRAMMS) checklist

| Guideline | Reported |
| --- | --- |
| Describe the justification for using mixed methods approach to the research question | The research question (exploring the multi-dimensional TB of stroke patients and its associated factors) has the characteristics of "complexity" and "multi-dimensionality". A single quantitative method can only verify the correlation between variables but cannot capture the subjective experience and contextual background of patients; a single qualitative method can explore in-depth experiences but lacks generalizable statistical evidence. The mixed methods approach (convergent parallel design) integrates the advantages of both: quantitative research provides objective evidence for associated factors, and qualitative research supplements the actual experience and mechanism interpretation of TB, thereby achieving a more comprehensive, accurate, and in-depth response to the research question. This justification is systematically presented in the Background, Method, and Aim sections of the document, and verified through the integration of results in the Discussion section. |
| Describe the design in terms of purpose, priority and sequence of the methods | This study adopts a convergent parallel mixed-methods design to explore the associated factors of TB in stroke patients. The quantitative and qualitative components were accorded equal priority: the former aimed to explore the distribution characteristics and associated factors of TB, while the latter focused on investigating patients’ subjective experiences of TB. These two strands of data were complementary; by integrating objective statistical results with in-depth contextual insights, they collectively addressed the research questions. |
| Describe each method in terms of sampling, data collection and analysis | In Sections 3.3 Data Collection and 3.4 Data Analysis of the manuscript, specific contents of the three dimensions (sampling, data collection, and data analysis) are elaborated in detail for both quantitative and qualitative research respectively.  Quantitative: Convenience sampling of 237 hospitalized stroke patients; data collected via 5 standardized scales and a general information questionnaire; analyzed using univariate analysis, correlation analysis, and multiple linear regression.  Qualitative: Purposive and maximum variation sampling of 17 patients; data collected via face-to-face semi-structured interviews; analyzed using Colaizzi’s seven-step method with Nvivo 11 software, supported by researcher triangulation. |

Supplementary material 1 Continued

| Describe where integration has occurred, how it has occurred and who has participated in it | Where: Integration occurred during data analysis and result presentation.  How: Two strategies—triangulation (cross-validating quantitative and qualitative results) and sequential integration (using qualitative insights to interpret quantitative trends)—with a joint display table (Table 6) visualizing confirmation, complementarity, and expansion.  Who: The entire research team participated in coding, discussion, and result synthesis. |
| --- | --- |
| Describe any limitation of one method associated with the present of the other method | Quantitative limitation: Self-report bias and insufficient assessment of time/energy investment in self-management behaviors.  Qualitative complementarity: Captured subjective experiences that quantitative data could not fully reflect, offsetting superficial measurements. |
| Describe any insights gained from mixing or integrating methods | Validated the Cumulative Complexity Model’s applicability in Chinese stroke patients, confirming depression, self-management efficacy, age, objective support, and dietary control as key associated factors.  Revealed the dynamic imbalance between patient capacity and workload as the core mechanism of treatment burden, providing actionable strategies (psychological interventions, individualized rehabilitation) for clinical practice. |

Supplementary material 2 Description of independent variable assignment

| Variable | Description of the assignment |
| --- | --- |
| Age | <45=1; 45~59=2; 60~74=3; ≥75=4 |
| Work status | Unemployed (0,0,0,0); Sick leave (0,1,0,0); On duty (0,0,1,0);  Retired（0,0,0,1） |
| Per capita monthly household income (CNY) | ≤2000=1; 2001~3000=2; 3001~4000=3; ＞4000=4 |
| Medical payment method | Medical insurance/public funds=0; Self-funded=1 |
| Type of stroke | Ischemic (0,0,0); Hemorrhagic (0,1,0); Both (0,0,1) |
| Primary caregiver | Parents (0,0,0,0,0); Spouse (0,1,0,0,0); Child (0,0,1,0,0);  Carer (0,0,0,1,0); Relative or other (0,0,0,0,1) |
| Duration of illness (months) | <6=0；≥6=1 |
| Number of sequelae | None=1; 1 type=2; 2 types=3; ≥3 types=4 |
| Co-morbidities with chronic diseases | None=1; 1 type=2; 2 types=3; ≥3 types=4 |
| Number of types of oral medications taken per day | ≤2 types=1; 3~5 types=2; ≥6 types=3 |
| ADL degree | Severely dependent (0,0,0,0); Moderately dependent (0,1,0,0);  Mildly dependent (0,0,1,0); Self-care (0,0,0,1) |
| The self-management efficacy | Measured value |
| The activities of daily living efficacy | Measured value |
| Dietary control | Measured value |
| Medication adherence | Measured value |
| Emotional management | Measured value |
| Rehabilitation exercise | Measured value |
| Smoking and alcohol cessation | Measured value |
| Objective support | Measured value |
| Social support utilization | Measured value |
| PHQ-9 | Measured value |

Supplementary material 3 Multiple linear regression analysis of TB in stroke patients

| Variable | *β* | *SE* | *β'* | *t* | *P* | 95% CI | | TOL | VIF |
| --- | --- | --- | --- | --- | --- | --- | --- | --- | --- |
|  |  |  |  |  |  | Lower | Upper |  |  |
| *β_0_* | 102.517 | 9.703 |  | 10.565 | <0.001 | 83.499 | 121.535 |  |  |
| PHQ-9 | 1.309 | 0.209 | 0.431 | 6.250 | <0.001 | 0.899 | 1.719 | 0.369 | 2.713 |
| The self-management efficacy | -0.286 | 0.144 | -0.235 | -1.995 | 0.047 | -0.568 | -0.004 | 0.126 | 7.910 |
| Age | -3.551 | 1.005 | -0.213 | -3.532 | 0.001 | -5.521 | -1.581 | 0.481 | 2.081 |
| Objective support | -1.702 | 0.515 | -0.171 | -3.302 | 0.001 | -2.711 | -0.693 | 0.651 | 1.537 |
| Dietary control | -2.008 | 0.607 | -0.163 | -3.307 | 0.001 | -3.204 | -0.812 | 0.720 | 1.389 |
| Sick leave | 3.369 | 2.496 | 0.069 | 1.350 | 0.179 | -1.551 | 8.289 | 0.645 | 1.551 |
| On duty | 3.841 | 2.116 | 0.104 | 1.815 | 0.071 | -0.331 | 8.013 | 0.513 | 1.948 |
| Retired | 2.141 | 1.964 | 0.072 | 1.090 | 0.277 | -1.732 | 6.013 | 0.389 | 2.568 |
| Per capita monthly household income (CNY) | -0.675 | 0.743 | -0.057 | -0.909 | 0.364 | -2.140 | 0.789 | 0.426 | 2.346 |
| Medical payment method | 0.158 | 2.549 | 0.003 | 0.062 | 0.951 | -4.867 | 5.182 | 0.820 | 1.219 |
| Hemorrhagic | 1.909 | 1.423 | 0.070 | 1.342 | 0.181 | -0.896 | 4.713 | 0.627 | 1.595 |
| Both | 0.857 | 2.540 | 0.015 | 0.337 | 0.736 | -4.151 | 5.865 | 0.826 | 1.211 |
| Spouse | 2.427 | 3.022 | 0.091 | 0.803 | 0.423 | -3.531 | 8.386 | 0.132 | 7.560 |
| Child | 0.496 | 3.494 | 0.016 | 0.142 | 0.887 | -6.392 | 7.384 | 0.140 | 7.156 |
| Carer | 0.245 | 3.413 | 0.006 | 0.072 | 0.943 | -6.484 | 6.974 | 0.252 | 3.970 |
| Relative or other | -2.062 | 3.840 | -0.034 | -0.537 | 0.592 | -9.632 | 5.509 | 0.418 | 2.393 |
| Duration of illness (months) | -0.484 | 1.384 | -0.018 | -0.350 | 0.727 | -3.212 | 2.245 | 0.669 | 1.495 |
| Number of sequelae | 1.381 | 0.776 | 0.091 | 1.780 | 0.076 | -0.148 | 2.910 | 0.645 | 1.550 |
| Co-morbidities with chronic diseases | 0.116 | 0.702 | 0.008 | 0.165 | 0.869 | -1.268 | 1.500 | 0.700 | 1.428 |
| Number of types of oral medications taken per day | -1.088 | 1.025 | -0.054 | -1.061 | 0.290 | -3.108 | 0.933 | 0.660 | 1.515 |
| Moderately dependent | -0.111 | 1.743 | -0.004 | -0.064 | 0.949 | -3.548 | 3.326 | 0.561 | 1.781 |
| Mildly dependent | 2.543 | 1.911 | 0.092 | 1.331 | 0.185 | -1.225 | 6.310 | 0.353 | 2.836 |
| Self-care | 2.468 | 2.883 | 0.069 | 0.856 | 0.393 | -3.217 | 8.152 | 0.265 | 3.779 |

Supplementary material 3 Continued

| The activities of daily living efficacy | 0.181 | 0.125 | 0.149 | 1.447 | 0.149 | -.066 | .428 | 0.161 | 6.214 |
| --- | --- | --- | --- | --- | --- | --- | --- | --- | --- |
| Medication adherence | -0.352 | 0.493 | -0.042 | -0.715 | 0.475 | -1.324 | 0.619 | 0.503 | 1.989 |
| Emotional management | -0.619 | 0.366 | -0.121 | -1.689 | 0.093 | -1.341 | 0.104 | 0.328 | 3.046 |
| Rehabilitation exercise | -.007 | 0.339 | -0.001 | -0.021 | 0.983 | -0.675 | 0.661 | 0.388 | 2.579 |
| Smoking and alcohol cessation | 0.594 | 0.431 | 0.061 | 1.379 | 0.169 | -0.255 | 1.444 | 0.856 | 1.168 |
| Social support utilization | -0.841 | 0.487 | -0.094 | -1.728 | 0.086 | -1.800 | 0.119 | 0.568 | 1.760 |

Note: *F*=13.225, *P*<0.001; *R*^2^=0.648; adjusted *R*^2^=0.599.
